# A global survey addressing sustainability of pollen monitoring^☆^

**DOI:** 10.1016/j.waojou.2024.100997

**Published:** 2024-11-19

**Authors:** Divya Dwarakanath, Andelija Milic, Paul J. Beggs, Darren Wraith, Janet M. Davies

**Affiliations:** a School of Biomedical Science, Faculty of Health, Queensland University of Technology, Brisbane, Queensland 4059, Australia; b School of Natural Sciences, Faculty of Science and Engineering, Macquarie University, Sydney, New South Wales 2109, Australia; c School of Public Health and Social Work, Faculty of Health, Queensland University of Technology, Brisbane, Queensland 4059, Australia

**Keywords:** Pollen monitoring, Aerobiology, Allergy, Sustainability

## Abstract

**Background:**

Contemporary airborne pollen records underpin environmental health warnings, yet how pollen monitoring networks are sustained is poorly understood. This study investigated by whom and how pollen monitoring sites across the globe are managed and funded.

**Methods:**

Coordinators listed in the Worldwide Map of Pollen Monitoring Stations were invited to complete a digital questionnaire designed to survey the people and organisations involved, types, and duration of funding sources, as well as uses, purpose, and sharing of pollen information. Quantitative data were analysed by descriptive statistics and open text responses were examined by qualitative thematic analysis.

**Results:**

Eighty-four of 241 (35%) coordinators from 37 countries responded. Universities (42%) and hospitals/health services (29%) were most commonly responsible for monitoring. Most sites involved employees (87%) in pollen monitoring, of whom many were part-time (41%) or casual (11%), as well as students (29%) and volunteers (6%). Pollen monitoring was additional to core duties for over one-third of sites (35%), and 25% reported pollen monitoring was an in-kind contribution. Whilst funding for pollen monitoring was often sourced from government agencies (33%), government research grants (24%), or non-government grants (8%), 92% reported more than 1 funding source, and 99% reported dependence on “partnerships or grants requiring co-contributions”, indicating a complex resourcing structure, of short duration (median 3 years). Common reasons why airborne pollen was monitored included clinical allergy, population environmental health, aerobiology and forecasting. Climate change, research, and social duty were also referenced.

**Conclusions:**

Aerobiological monitoring is currently sustained by complex, insecure, and insufficient resourcing, as well as reliance on volunteerism. There are multiple direct, health-related, and other important uses of aerobiology data, that are aligned to multiple dimensions of sustainability. Evidence from this study can be used to inform the design of strategies to sustain the generation of aerobiology data.

## Introduction

The monitoring of airborne pollen aligns with several United Nations Sustainable Development Goals (SDGs) including, as examples, SDG 3 Good Health and Wellbeing, SDG 13 Climate Action, and SDG 15 Life on Land.^1^ Whilst the global prevalence and burden of disease due to asthma affecting over 300 million people is extensive and well documented,^2^ the total burden of pollen allergies including allergic rhinitis (AR) is less clear, possibly due to inconsistency of disease coding and analysis,^3^ complexity of direct and indirect costs,^4^ presence of various comorbidities,^5^ and lack of access to medical care by AR patients.^6^ A major motivation for monitoring airborne pollen is the provision of local pollen information to help patients with allergic respiratory disease avoid pollen exposure, and to help clinicians with management of AR and asthma patients.

Allergy patients often seek local daily information and forecasts indicating airborne pollen exposure which are made available by pollen monitoring sites via mobile apps and online pollen website platforms^7^ to enable self-management of medication use and allergen avoidance strategies.^8^^,^^9^ Other important health-related uses of airborne pollen data encompass investigating thresholds of exposure associated with allergy symptoms,^10^ analysis of symptom reduction during clinical trials of allergen immunotherapy,^11^ inclusion in epidemiological studies,^12^ and analyses with physical and chemical weather including gaseous and particulate pollutants.^13^

As well as the perceived community need for access to timely standardized pollen exposure information,^14^, ^15^, ^16^ there is an expectation by healthcare services and other stakeholders, that airborne pollen monitoring data are accurate,^17^^,^^18^ pollen forecasts are based on monitoring observations,^19^^,^^20^ and that the forecasts are evaluated and validated.^21^^,^^22^

Mounting evidence indicates that climate change, including increasing temperatures and concentrations of CO_2_, landscape modifications, urbanisation, and invasive species migration, contribute to trends in seasonal airborne pollen loads and complexity.^23^^,^^24^ Climate change is expected to continue to drive increases in exposures to pollen allergen sources,^25^^,^^26^ meaning ongoing, standardised monitoring of airborne pollen levels will remain important for documenting evidence of medium- to long-term trends in local airborne pollen concentrations across all regions of the world.^27^

In 2018, Buters et al published information on 879 active aerobiological monitoring stations globally, of which the majority were in the Northern Hemisphere (68%): Europe (525), North America (134), and Japan (120).^28^ At that time, 70% of pollen monitoring networks globally relied on the Hirst-type volumetric continuous flow impaction pollen monitoring trap. Although the Hirst-type pollen traps are robust, sample collection and microscopic counting are labour intensive, and reliance on manual methods is an unsustainable approach to monitoring. Whilst application of automated pollen monitoring has gained momentum,^29^ the devices are not always successful at distinguishing different taxa,^30^ and there is much still to learn about their operation in practice, and comparability between automatic and traditional monitoring methods.^31^ Nonetheless, requirements and placements of automated pollen monitoring sites for European networks are being considered,^32^ and automated pollen monitoring is well advanced in Japan.^33^ During the transition to automated pollen monitoring it is recommended that there is at least a five-year overlap to train and verify locally the performance of automated pollen monitoring devices and their suitability for monitoring pollen from locally relevant taxa.^34^ In regard to equitable and sustainable development,^35^ it should be noted that high-precision automated pollen monitors are expensive; in the order of 50–100 times the cost of Hirst-style pollen trap,^36^ and may present a financial barrier to their installation, operation and maintenance.

Despite the perceived importance of aerobiological monitoring data, the ways in which pollen monitoring activities are sustained by networks across the globe has not been purposely investigated. Lessons learnt from experiences with coordinating pollen monitoring in different regions of the world may inform strategies for both well-established and emergent pollen monitoring networks, particularly those in the Southern Hemisphere,^37^ to sustain pollen monitoring activities worldwide. The aim of this study is to investigate the ways in which airborne pollen monitoring sites are managed and funded across the globe. Outcomes of this study should provide insights to develop sustainable approaches to delivery of continuous airborne pollen monitoring data.

## Methods

### Study and questionnaire design

The primary research question “How are pollen monitoring sites managed and funded?” was addressed using a purpose designed survey to identify the characteristics, management, and funding of pollen monitoring networks globally. An issue tree was used to understand the structure of pollen monitoring processes to identify the system components that required attention in the Sustaining Pollen Monitoring (SPM) survey design. The necessary components for pollen monitoring included: the pollen monitoring instrument type, organisational management, pollen monitoring processes, pollen information dissemination, and human and financial resources. There were 29 survey items that mapped to the following issues: a) the types of organisations and networks that manage pollen monitoring, b) the employment status of people who operate the pollen monitoring station, c) funding sources for operating pollen monitoring stations, d) purposes, dissemination, and uses of pollen information, and e) issues identified by people who coordinate pollen monitoring stations.

The pilot version of the questionnaire was tested and refined at a national aerobiology workshop in 2019, with 19 participants from academic and health service agencies involved in pollen monitoring. No data were retained from the pilot version. The questions were revised according to workshop feedback prior to survey distribution and data collection. Where appropriate, the choice to respond “I don't know” or “Prefer not to answer” was provided. Individuals could also choose not to answer a question, and in some cases, tick all response options that applied. The number of responses to each question was recorded and reported. Participant response data were collected through the SPM questionnaire (Supplemental appendix - Questionnaire) administered via KeySurvey.

### Study population and questionnaire distribution

This study was approved by the University Human Research Ethics Committee. Primary survey data is not available for sharing for ethical and privacy reasons since participants could be re-identified in the deidentified survey data because of the small numbers of people who coordinate pollen monitoring in each country. The study population included individuals named as the contact person for pollen monitoring stations with an email address publicly available and accessed (April 2020) on the pollen monitoring map website^28^ (see supplementary material regarding inclusion of stations). Email addresses for coordinators of the pollen monitoring networks who were listed as site contacts were extracted. Duplicate email addresses were removed, and the retrieved email addresses were divided into contacts from the Northern and Southern Hemispheres so the survey could be distributed prior to or during the relevant spring-summer pollen seasons. An invitation to participate, with a link to the study information and questionnaire, was emailed and two reminders were sent, one two weeks after the first, and the second a week before the relevant closing date. The invitation to participate was also included in a newsletter published by the International Aerobiology Association. To ensure timely response within the relevant season, participants were given 30 days to complete the survey. The invited coordinators of the pollen monitoring stations gave informed electronic consent to participate after clicking the embedded link to the questionnaire and reading study information.

### Data analysis

Numerical and categorical responses were examined using descriptive statistical analyses in Graphpad Prism (version 10, GraphPad Software, Massachusetts, USA). Normality of continuous numerical data was assessed by the Kolmologorov-Smirnov test. Distributions of skewed data were reported as median and interquartile range (IQR). Open text responses given where there was an option “other” in categorical questions, were either converted into numbers, or reported in quantitative analysis as “other”. Text responses to the open-ended questions were coded using NVIVO 14 (Lumivero, Colorado, USA). Deduced thematic analyses^38^ were performed by two researchers and moderated by another.

## Results

### About the survey participants

**Fig. 1:**
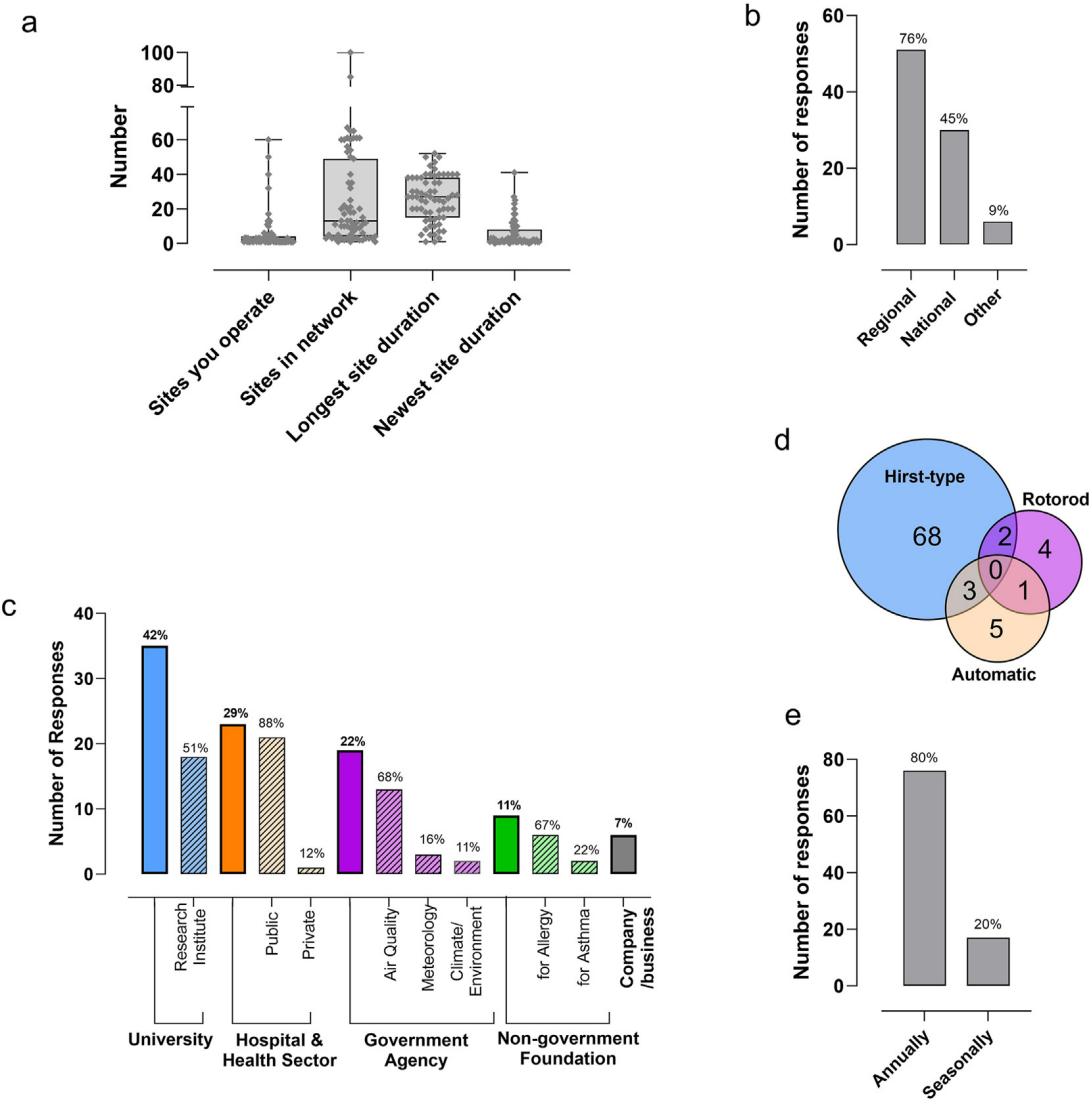
Features of pollen monitoring networks coordinated by study participants. a) the number and duration of sites operated, b) number and percent of involvement in regional and/or national networks, c) number and percent of responses by type of organisation managing sites (solid bars), with number and percent of sub-options shown (light hashed bars), d) type(s) of instrument used, and e) period of monitoring

Of 879 listed pollen monitoring stations, email addresses were retrieved for 632, of which 263 were unique. Of 263 invitations sent, 22 were undelivered, and three recipients declined to participate. A total of 84 (ie, 35% of 241 pollen monitoring site coordinators who received an invitation) commenced and completed the questionnaire through to penultimate question, the last question being optional. Participants represented 37 countries (Supplemental Fig. 1) and reported operating a median of 2 pollen monitoring stations (IQR 1–4) with the maximum being 60 (Fig. 1A).

### Inclusion in pollen monitoring networks

Sixteen responded they were not part of a pollen monitoring network. Of 67 (80%) of participants who indicated being part of a network, 51 (76%) belonged to national, and 30 (45%) belonged to a regional network (Fig. 1B). Fourteen participants indicated being part of both a regional and national network. The median number of stations in the networks was 13 (IQR 4–49) (Fig. 1A). Overall, the longest running station was 52 years and the newest was one month, with the median longest and shortest reported station durations within a network being 27 years (IQR: 15–38) and 2 years (IQR: 1–8), respectively (Fig. 1A).

### Operation of pollen monitoring stations

The highest number of pollen monitoring stations were operated by universities (*n* = 35, 42%, Fig. 1C), with 18 being in the sub-option "research institute". The second highest option selected was hospital and health sector (*n* = 24, 29%), predominantly in the sub-option "public hospitals services" (*n* = 21, Fig. 1C). The third highest option recorded was "government agency" (*n* = 19, 22%), 13 of which were air quality and 3 were meteorology service providers. Six participants (7%) reported that pollen monitoring stations were managed by a private company or business, and 9 (11%) reported that stations were run by non-government foundations such as associations for clinical immunology and allergy, or asthma.

Of the 83 participants who responded to the question regarding instruments used, the most commonly reported type was the Hirst-type pollen trap (*n* = 73, 89%), followed by automated pollen monitor (*n* = 10, 11%), and Rotorod pollen trap (n = 7, 8%). However, 6 (7%) of these participants reported using a combination of 2 device types (Fig. 1D). Aerobiological monitoring was conducted more often over the full duration of the year (*n* = 67, 80%) than just within the pollen season (*n* = 17, 20%; Fig. 1E).

### The people who monitor pollen

**Fig. 2:**
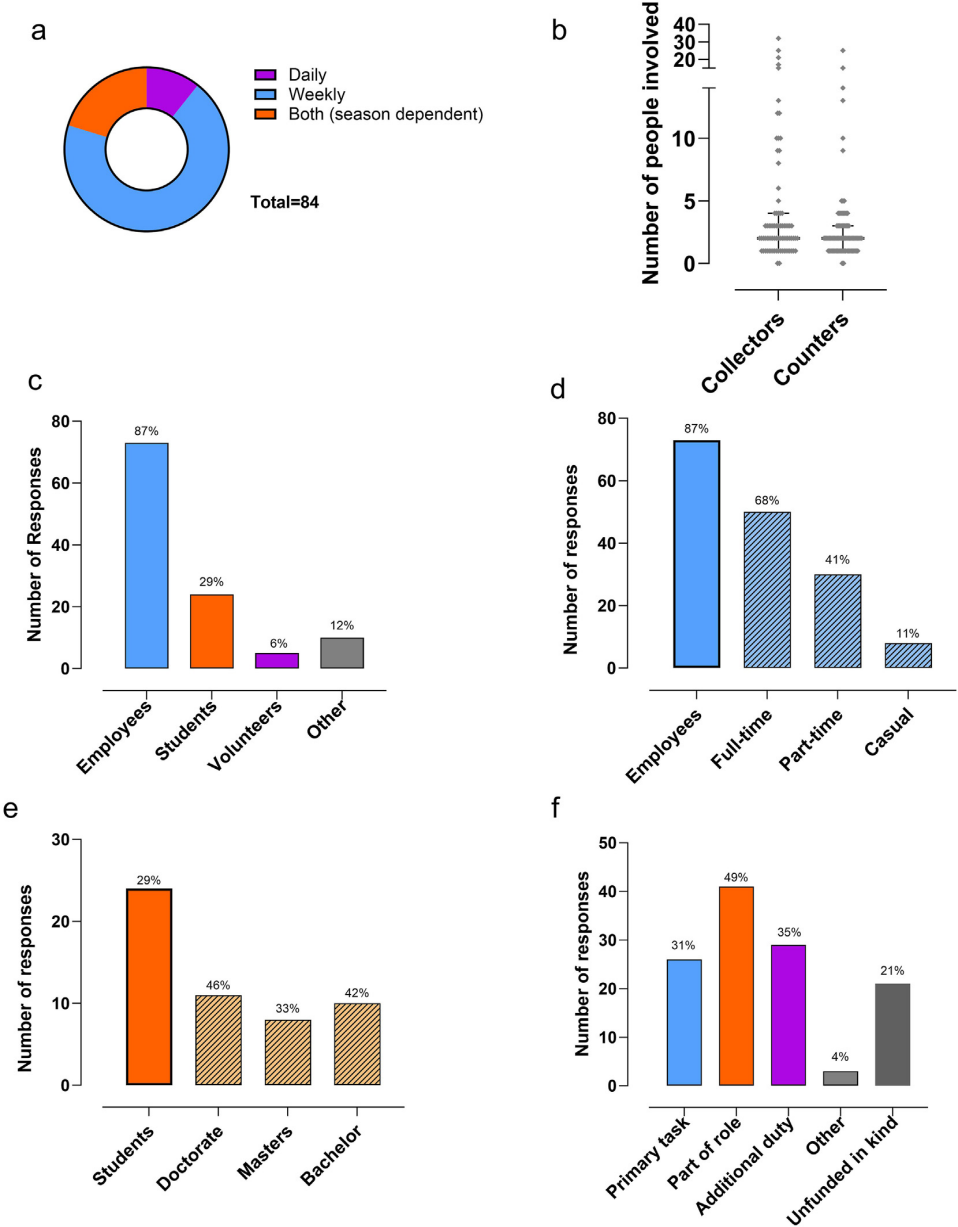
Distribution of workload of involved in pollen monitoring. a) frequency of sample collection from site, b) number of people involved as sample collectors and pollen counters, c) number and percent of people involved by role, d) for sites involving employees (solid colour), the number and percent of staff employed by status (light hashed bars), e) for sites involving students (solid colour) the number and percent of students by degree type (light hashed bars), and f) role of pollen monitoring with respect to usual duties of people involved (number and percent of responses).

Fifty-eight (70%) participants reported visiting their pollen monitoring stations weekly to collect samples, and 9 (11%) reported collecting pollen samples daily (Fig. 2A). A further 17 (20%) participants collected pollen samples daily or weekly, depending on the season.

Nineteen participants (24%) responded that 1 person collects the sample from the trap, and 57 (76%) participants reported having more than 1 person to do this task. Likewise, 23 participants (28%) indicated that they had 1 person identifying and counting pollen, whilst 56 (72%) participants responded that more than 1 person assisted with pollen identifying and counting. The median numbers of people involved in collecting, and identifying and counting, pollen respectively were 2 (IQR 1–4) and 2 (IQR 1–3) (Fig. 2B).

The majority of participants responded that employees (*n* = 73, 87%) contributed to pollen monitoring duties (Fig. 2C). Of these, 50 (68%) responded that staff were appointed full-time, part-time (30; 41%) and/or casually (8; 11%), with multiple responses indicating a mixed workforce composition (Fig. 2C). Twenty-four (29%) responded that the counters included students, with similar numbers undertaking PhD, Masters and Bachelor degrees (Fig. 2D). Five respondents indicated people contributing to pollen monitoring activities were volunteers. Of those who responded “other”, several explained they “do not need” counters because they used “automatic pollen monitors”, and others indicated “residents”, “medical allergologists” or “allergists from hospitals where collectors are located”.

Twenty-six (31%) participants responded that pollen monitoring was the primary task, whilst for 41 (49%) respondents pollen monitoring was part of the usual role, and 29 (35%) indicated that this activity was additional to core duties of the pollen counters. Twenty-one (25%) indicated pollen monitoring was an unfunded, in-kind, activity.

### Dissemination and research use of pollen information

**Fig. 3:**
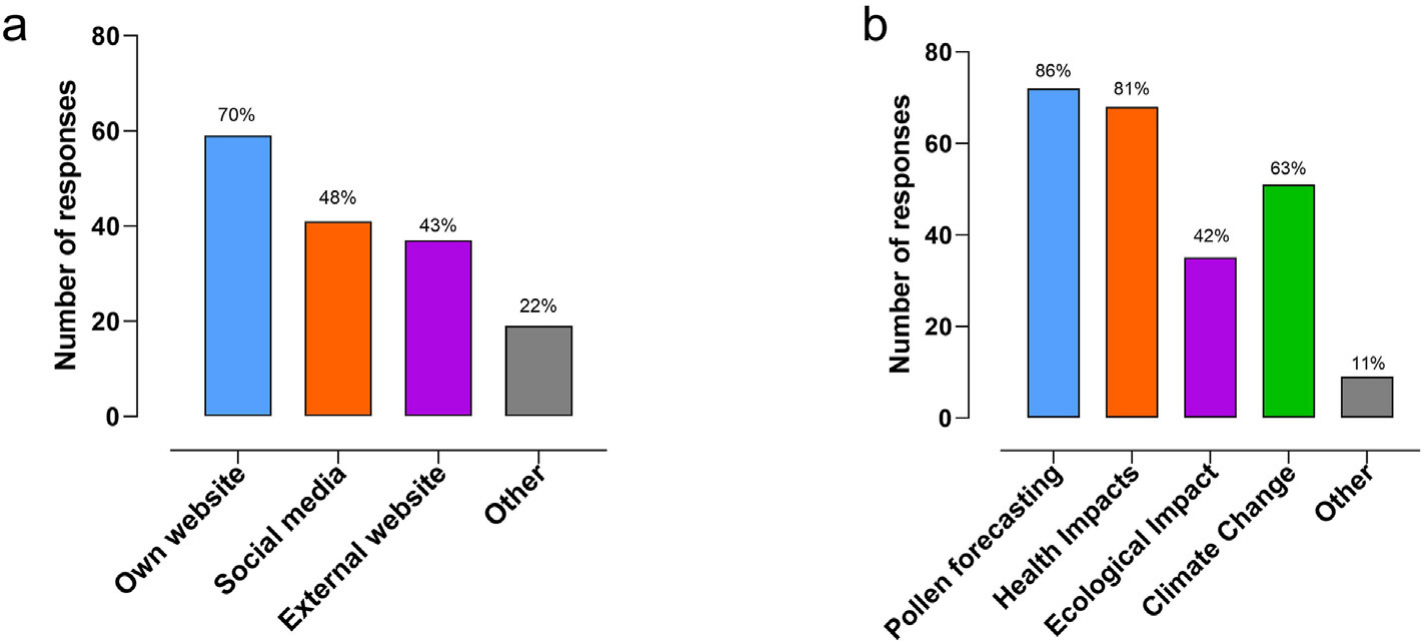
Dissemination and uses of pollen monitoring information. a. routes of sharing pollen information, and b. research uses of airborne pollen data. Respondents (n = 84) selected multiple response options. Data shown as number and percentages of responses

Forty-one (49%) participants reported sharing pollen information daily during the pollen season. However, 58 (69%) participants provided pollen information through their own organisation website, but not necessarily on a daily basis (Fig. 3A). The second highest daily dissemination route was via social media apps (n = 40, 48%), followed by external organisation platforms (n = 36, 43%) such as foundations for asthma patients. Other reported ways of sharing pollen information included media platforms such as radio or newspapers, non-government foundation websites, booklets, or brochures.

Regarding research use of airborne pollen data, multiple research purposes were indicated by most of the 84 respondents; 72 (86%) indicated pollen forecasting, 68 (81%) indicated health impacts, 51 (63%) indicated climate change, and 35 (42%) indicated ecological impact studies (Fig. 3B). The other responses included research on phytopathology, forensic palynology, agronomy, forestry, and environmental pollution. The number of pollen and spore types counted and pollen types considered to have most impact on human health are presented in Supplementary Fig. 2.

### Funding sources for sustaining pollen monitoring

**Fig. 4:**
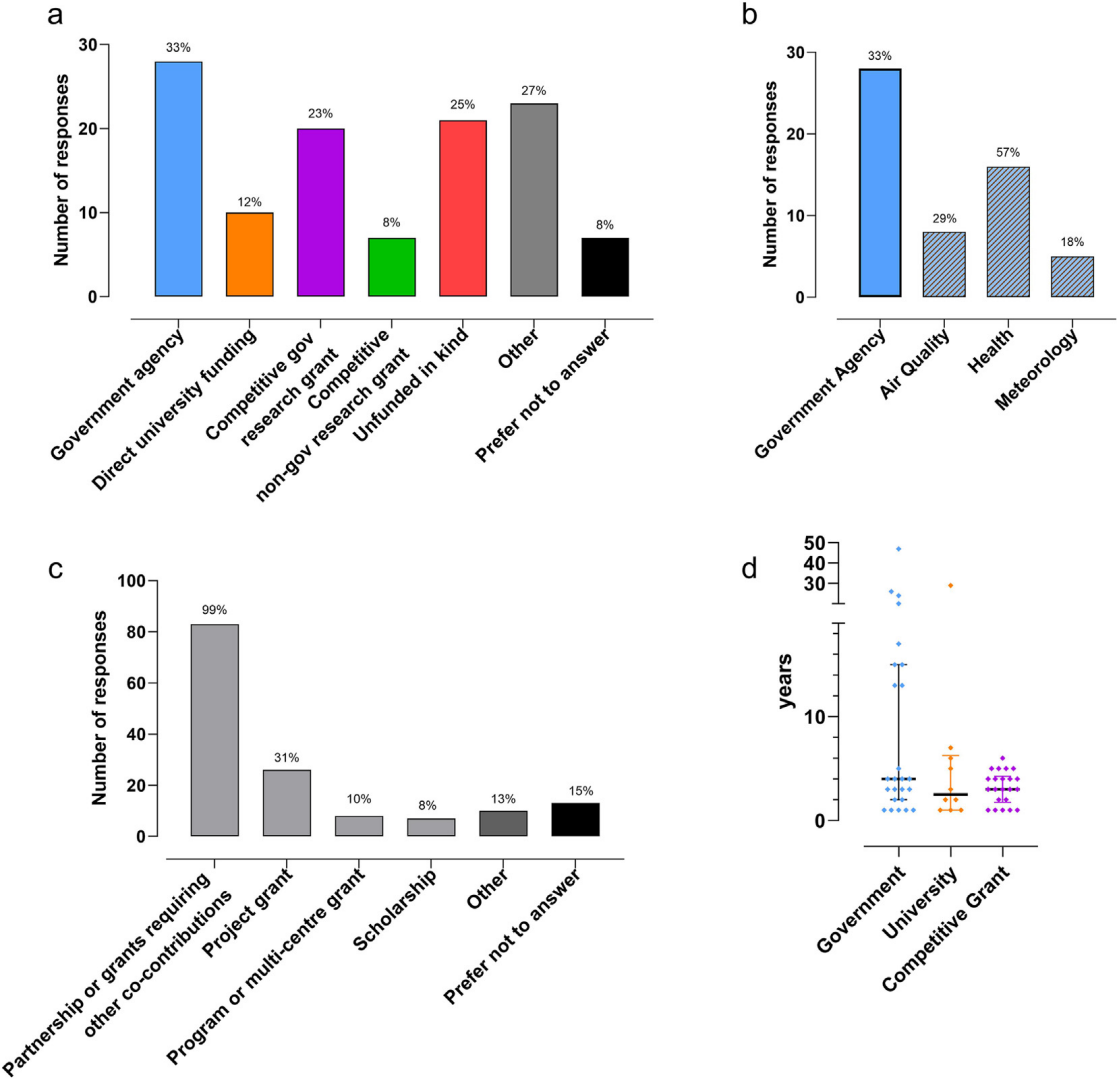
Funding to support pollen monitoring activities. a) number and percent of responses receiving various sources of funding, b) for sites with government funding (solid colour), the number and percent by agency (light hashed bars), c) types of grants supporting pollen monitoring (number and percentage of responses), and d) duration of funding from different sources. Respondents (n = 84) selected multiple response options.

Twenty-eight (33%) of the pollen monitoring networks were supported via government agency funding (Fig. 4A). The sub-options indicated that a majority were health departments (*n* = 16, 19%, Fig. 4B). The next common funding source was competitive government research grants (n = 20, 23%). Nearly all respondents indicated that they received funding from “Partnership or grants requiring co-contributions from other sources” (n = 83, 99%) (Fig. 4C). The other responses (n = 22, 27%) included scientific and clinical societies (eg, allergology and clinical immunology), private clinics, consulting or private companies, local or regional government (councils), allergy and immunology centres, and “sales of pollen data to media and commercial research”. Twenty (25%) of respondents indicated that pollen monitoring was “Unfunded, in-kind contribution” (Fig. 4A).

The median durations of competitive research grant funding and university funding were 3 years (IQR; 1.75–4.25) and 2.5 years (IQR 1–6.25), respectively (Fig. 4D). Whilst government funding (4 years; IQR 2–15 years) tended to be slightly longer than competitive grant or university funding, these differences in duration were not significant (Fig. 4D).

### Reported reasons for monitoring airborne pollen

The overarching themes of clinical allergy and environmental public health, with subthemes related to supporting health and medical services, and supporting allergy patients, were the dominant reasons why airborne pollen was monitored (Supplemental Table 1). Whilst some reasons related to health protection or population level disease prevention, more direct clinical use for individual diagnosis and treatment decisions were also mentioned.

“We monitor pollen as part of our Program of preventive action on health protection.”

"To improve the diagnosis and treatment of patients with pollinosis."

“To protect human health by giving allergy sufferers the knowledge they need to minimise exposure.”

The concept of pollen monitoring being “a service to persons suffering from pollen allergies” was referred to. A more explicit theme related to “social duty”, and how duty changed and extended beyond the bounds of one's own, “my research project”.

“Climate change”, “airborne pollen information”, and “research”, including biodiversity, invasive plants, fungal spores and air pollution, were also important reasons referenced multiple times.

“… evaluation of the effects of climate change on the beginning and end of flowering and the quantities of pollen …”

In open feedback, resourcing and financial considerations, training and data quality, research, and collaboration were the dominant themes (Supplemental Table 2).

Tied to the theme of service, responses to this subsequent question conveyed belief in the value or worth of pollen monitoring, evoking a sense of altruism for undertaking the activity.

“We believe that every effort to continue with the monitoring network is excellent and worthwhile …”.

Financial constraint was referenced as a reason for cessation of pollen monitoring or loss of staff in three responses (Supplemental Table 2). There was also a sense of unmet expectation that government should have a role in funding pollen monitoring;

"Sustainability is a major issue in <country> as governments typically do not see sustaining pollen monitoring as core business …”

Costs and maintenance were recognised as necessary for both automated and traditional monitoring approaches.

“investments in pollen measurement can continue to be justified (for monitoring) automatically or traditionally."

" … The type of climate and other environmental factors in the country also hastens the "wear and tear" of the pollen trap used …."

As well as a lack of funding, administrative load of managing multiple funding sources was acknowledged.

“… the main difficulty is dealing individually with each of the parties for many different aspects: the annual reporting, negotiating the amount and duration of grants, responding to specific requests, etc.”

In relation to pollen information, a number of points relevant to pollen transport and representation of sites, were mentioned. Participants from Southern Hemisphere (n = 7) expressed additional concerns, such as “lack of collaborative research in the Southern Hemisphere”, “seeking global research opportunities”, “inconsistent funding”, and “maintenance of networks” (Supplemental Tables 1 and 2).

## Discussion

This study is the first to investigate sustainability of pollen monitoring networks globally. There appeared to be no previous directly relevant literature on this topic. When considering downstream usage of pollen monitoring data and requirements for optimal monitoring networks, Sofiev and European colleagues, assert in relation to density and location of network sites that “the limitations are purely financial”,^32^ reinforcing the need to better understand resourcing of pollen monitoring. Our findings indicate that there is a need for analysis of issues related to funding stability because this financial risk to continuation of pollen monitoring remains relevant and important, regardless of any transition towards automated systems. Sustained pollen monitoring is a necessary requirement for long pollen records to track effects of climate change on pollen exposure,^23^^,^^25^ integration of contemporary airborne pollen records with other palynological datasets,^39^ and interaction of bioaerosols with other meteorological parameters for health impact assessments.^13^

A study limitation was the lack of a validated research tool to apply. However, the SPM questionnaire was tested by a leading national aerobiology collaboration network (AusPollen),^40^ and the consensus was that the structure of the questionnaire was useful to assess the complexities of sustaining a pollen monitoring network or station. Whilst there was greater participation from Northern Hemisphere than Southern Hemisphere, this is consistent with the distribution of pollen monitoring sites across the globe,^28^ and discontinuous or emergent pollen monitoring networks; South African Pollen Network (SAPNET), South American (Argentina), and Australian continents.^41^, ^42^, ^43^ The survey attracted a reasonably large (35%) sample of known pollen monitoring site coordinators, but notably there were few participants from Asian countries, which may be partly explained by survey being only available in English. Coordinators of sites not included in the worldwide map of pollen monitoring may not have had opportunity to contribute to this survey.

It is important to consider that this study found that universities were involved in operating 42% of pollen monitoring stations. Whilst there may be a directive in some jurisdictions, like Europe, for active dissemination of environmental information,^44^ this may not apply to academic settings. Notably, the core academic priority of knowledge generation, measured by high impact scientific publications,^45^ carries more value for researchers than routine generation of pollen monitoring datasets. Whilst the value, equity and complexities of open data sharing are now well appreciated,^46^ sharing of pollen monitoring data *per se* may not be a highly weighted academic output.^47^

Government agencies, which have a core responsibility to monitor weather and/or air quality, underpinned pollen monitoring services in only 23% of responses. Whilst most pollen monitoring networks recommend standardised procedures and methodologies for pollen sampling to ensure reproducible and comparable data,^14^^,^^15^^,^^36^ themes relating to training, standardization and quality, indicate that data quality remained a concern amongst participants. Close inter-sector partnership or collaborations between academia and government agencies might offer benefits of innovation from the academic sector and rigour of quality systems operational for other routine government monitoring services.

That universities were the dominant contributor to pollen monitoring activities carries financial risk to sustained pollen monitoring because of the intrinsic competitive nature of research grant funding schemes for which outcomes are subject to the vagaries of peer review processes.^48^ Further drawbacks of reliance on uncertain competitive research grant funding for a large proportion of pollen monitoring are the short-term nature of project grants and influence by political or other factors on the scope of grants offered,^49^ or even funding decisions.^50^

There is evidence that pollen information has been largely consumed during the main pollen season especially during the season of high allergenic pollen species.^51^ However, generating long-term datasets over the entire year is useful for research purposes, and most participants (80%) indicated they monitor pollen all year round. If pollen is only monitored during known peak pollen seasons, then shifts in the start or end may be missed, which has been the case for some long-term pollen records for instance in Melbourne, Australia,^43^ reducing the utility and value of data for research re-use.

Most of the participants indicated that the pollen samples were collected and counted by more than one person, suggesting the workload was shared within or across small teams. Variability in numbers of people involved, frequency of site visits, and dissemination of pollen information, reflects flexibility in processes for collecting samples and counting pollen, and that timing of pollen counting can be uncoupled from dissemination of pollen information. Networks may have people who collect weekly samples at local or disbursed sites that are sent to a laboratory within the network for counting. Sites may share a daily pollen forecast that is verified by subsequent counting of samples collected weekly.

An important finding was that although over 87% of respondents indicated monitoring pollen involved employees, a major proportion were part-time (41%) or casual (27%). We were not able to collect data to indicate whether the seemingly high rates of part-time or casual work were consistent with workforce norms in each country. However, the involvement of employees working part-time to do pollen monitoring is well above the median rate for part-time work reported for 38 countries in the Organisation for Economic Co-operation and Development was 16.2% (IQR 9.3–18.9).^52^

It is also an important finding that a significant proportion of pollen monitoring activities are undertaken outside primary work duties, or beyond a researcher's own project, unfunded or conducted in some cases by volunteers. This finding is consistent with the present situation reported for the American National Allergy Bureau, which is “staffed primarily by the American Academy of Allergy Asthma and Immunology member volunteers who generously donate their time and expertise”.^36^ The sense of “worth” attributed to pollen monitoring information, and the “social duty” expressed, may each partly explain the willingness of staff, students and volunteers to spend unpaid or extra time on pollen monitoring activities. The equity and individual financial security of dependence on part-time or unpaid work for pollen monitoring workload warrants further sociological research beyond the scope of this study. From the perspective of sustainability, this workforce profile may also have implications and risk for continuity, training, skill retention, and quality of data because pollen monitoring appears to have a high dependence on the discretionary effort.

A core domain of sustainability for environmental health programs is funding stability since resourcing is crucial in the maintenance of quality services.^53^ That many respondents accessed multiple funding sources and grant types, suggests primary funding sources were insufficient to cover full costs of pollen monitoring. Networks or stations that draw from multiple diverse sources of funding may be more resilient.^54^ Access to multiple sources is known to help support consistency of health promotion programs.^55^ However, participants reported that accessing funding from diverse sources carried a high administrative burden, that may exacerbate workload and workforce instability. Experiences in other environmental science fields illustrate the opportunity costs and challenges of engaging external stakeholders, and the long lead time for securing research funding.^56^

Future strategic funding models^57^ must encompass in-depth understanding of resource requirements and benefits of pollen monitoring data, and establish partnerships with funders, in the context of complex systems.^57^ Moreover, the ethical role and risk of bias of for-profit entities, or sale of pollen monitoring data, in resourcing of pollen monitoring or provision of pollen information to community, health service or other stakeholders, warrants further considered. Access to resourcing to sustain monitoring activities depends on clear evidence of unmet need and political will. Whilst associations between pollen exposure and asthma hospital attendance are established,^12^ the burden on health systems outside the context of thunderstorm asthma^58^ due to pollen induced asthma may be less clear. The health impacts of pollen allergies should provide sufficient impetus for pollen information services to be publicly funded as an essential public health tool for management of AR and its comorbidities, including but not only asthma.^5^ Loss of quality of life and health costs of AR,^3^^,^^4^^,^^59^ should outweigh and justify the costs of pollen monitoring. Further social impacts of pollen allergies such as reduction of school leaver examination outcomes^60^ illustrate broader societal consequences of pollen allergies. However, without good quantitative evidence, locally or globally, of the true cost of pollen allergies, it is difficult to build a robust financial cost-benefit case for sustaining pollen monitoring as a properly resourced service.

As well as environmental health applications, which can be linked to SDG3; Good Health and Well-being, participants indicated multiple types of research uses of pollen data including biodiversity and climate change, consistent with other SDG; SDG 13 Climate Action, as outlined in an environmental pollen diversity assessment,^1^ suggesting a broader funding base may exist. The uneven continental impacts of climate change^26^^,^^61^ should be a compelling reason for networks across all continents to enable global scale analyses of spatiotemporal shifts in pollen concentrations. Collaborations with climate change researchers are beneficial when aerobiologists and environmental health researchers share a similar goal of supporting healthy communities.^62^ In the case of Australia, the pollen monitoring network was initiated by academic researchers, predominantly supported by high impact competitive research grant funding.^40^ Beyond research, the need for standardized pollen monitoring is recommended in the Royal Commission in Natural Disasters Report,^63^ but government support for pollen monitoring has been confined to the one state where epidemic level thunderstorm asthma is a threat,^21^ failing to address broader and seasonal needs of AR patients across all states. Since costs for AR are borne largely by the patient,^64^ government agencies may not be incentivised by cost savings to health services to allocate resources for pollen monitoring. Since aerobiology is a multidisciplinary endeavour, multiple lines of government responsibility may co-exist within health, meteorology, or environmental protection agencies, as reflected in this dataset. However, the spread of stakeholder interest across several agencies who might benefit, contribute to or support pollen monitoring, may obscure problem ownership, decision making and resourcing pathways.

## Conclusion

This study provides primary data from aerobiologists on the rationale for monitor pollen and promotes strategies using research to raise awareness of issues that encompass pollen monitoring. The challenges associated with sustaining pollen monitoring and pollen monitoring network management appear to be pervasive and multifaceted. There is an expressed environmental health need for timely airborne pollen data to underpin both daily and seasonal pollen forecasts for the purpose of reducing the burden of pollen allergies. However, there are also multiple multidisciplinary research uses of airborne pollen data. Sustaining pollen monitoring as a public health service, and/or for research, needs consideration of all core components of sustainability to be effective, and will need to be strategically planned and implemented in the changing contexts of transition to automated pollen monitoring capabilities.

## Author consent for publication

All authors have provided written permission to submit this manuscript to be considered for publication in the World Allergy Organization Journal.

## Availability of data and materials

The SPM survey tool is available in the Supplemental appendix – Questionnaire, which has been submitted with this manuscript. Primary survey data are not available for sharing for ethical and privacy reasons since participants could be re-identified in the deidentified survey data because there are only a small number of people who coordinate pollen monitoring in each country.

## Author contributions

All authors contributed to conception and design of the study. DD and JMD collected study data, and DD, AM and JMD performed data analysis. DD and JMD drafted the manuscript. All authors contributed to data interpretation, manuscript revision, and approved the submitted version of the manuscript.

## Ethics statement

The study was approved by the Queensland University of Technology Human Research Ethics Committee (approval number 1900000563/2518).

## Confirmation of unpublished work statement

The authors declare that this manuscript is original, has not been published before and is not currently being considered.

## Financial support

This research was supported by a Queensland University of Technology Postgraduate Scholarship from the Centre of Child Health Research, and a grant from the Australian National Health and Medical Research Council (NHMRC) for the AusPollen Partnership project (GNT1116107). The funders had no involvement in the study design; in collection, analysis, and interpretation of data; in the writing the manuscript; and in the decision to submit the article for publication.

## Declaration of competing interest

Related to this study, DD received support from Queensland University of Technology Postgraduate Scholarship from the Centre of Child Health Research, and JMD led the NHMRC AusPollen Partnership Project (GNT 1116107) with matching cash and in kind co-sponsorship from The Australasian Society for Clinical Immunology and Allergy, Asthma Australia, Bureau of Meteorology, Commonwealth Scientific and Industrial Research Organisation, Stallergenes Greer Australia, Federal Office of Meteorology and Climatology MeteoSwiss, Switzerland. In the last three years, JMD reports grants from Australian Research Council (ARC DP240203307 and DP210100347) outside the submitted work. JMD's institute QUT owns patents US PTO 14/311944 and a patent AU2008/316301 issued. PJB and JMD report loan from Kenelec Scientific of a Swisens Poleno Mars automated pollen monitor in 2023–2024. The other authors report no competing interests.

## References

[1] Hornick T., Richter A., Harpole W.S. (2022). An integrative environmental pollen diversity assessment and its importance for the Sustainable Development Goals. Plants People Planet.

[2] Global Burden of Disease Chronic Respiratory Diseases Collaborators (2023). Global burden of chronic respiratory diseases and risk factors, 1990-2019: an update from the Global Burden of Disease Study 2019. Clin Med.

[3] Wise S.K., Damask C., Roland L.T. (2023). International consensus statement on allergy and rhinology: allergic rhinitis-2023. Int Forum Allergy Rh.

[4] Blaiss M.S. (2010). Allergic rhinitis: direct and indirect costs. Allergy Asthma Proc.

[5] Bousquet J., Ansotegui I.J., Anto J.M. (2019). Mobile Technology in allergic rhinitis: evolution in management or revolution in health and care?. J Allergy Clin Immunol Pract.

[6] Katelaris C.H., Sacks R., Theron P.N. (2013). Allergic rhinoconjunctivitis in the Australian population: burden of disease and attitudes to intranasal corticosteroid treatment. Am J Rhinol Allergy.

[7] Bastl K., Bastl M., Bergmann K.C., Berger M., Berger U. (2020). Translating the burden of pollen allergy into numbers using electronically generated symptom data from the patient's hayfever diary in Austria and Germany: 10-year observational study. J Med Internet Res.

[8] Medek D.E., Simunovic M., Erbas B. (2019). Enabling self-management of pollen allergies: a pre-season questionnaire evaluating the perceived benefit of providing local pollen information. Aerobiologia.

[9] Bosnic-Anticevich S., Costa E., Menditto E. (2019). ARIA pharmacy 2018 "allergic rhinitis care pathways for community pharmacy": AIRWAYS ICPs initiative (European innovation partnership on active and healthy ageing, DG CONNECT and DG sante) POLLAR (impact of air POLLution on asthma and rhinitis) GARD demonstration project. Allergy.

[10] Kiotseridis H., Cilio C.M., Bjermer L., Tunsater A., Jacobsson H., Dahl A. (2013). Grass pollen allergy in children and adolescents-symptoms, health related quality of life and the value of pollen prognosis. Clin Transl Allergy.

[11] Hoffmann T.M., Acar Sahin A., Aggelidis X. (2019). "Whole" vs. "fragmented" approach to eaaci pollen season definitions: a multicenter study in six southern European cities. Allergy.

[12] Erbas B., Jazayeri M., Lambert K.A. (2018). Outdoor pollen is a trigger of child and adolescent asthma emergency department presentations: a systematic review and meta-analysis. Allergy.

[13] Klein T., Kukkonen J., Dahl A. (2012). Interactions of physical, chemical, and biological weather calling for an integrated approach to assessment, forecasting, and communication of air quality. Ambio.

[14] European Committee for Standardization (2019). https://standards.cen.eu/dyn/www/f?p=204:110:0::::FSP_PROJECT,FSP_ORG_ID:62260,6245&cs=195DA47915913BE03C00E5FDC54CD705F.

[15] Beggs P.J., Davies J.M., Milic A. (18 October 2018).

[16] National Allergy Bureau. National allergy Bureau: American Academy of allergy asthma and. Immunology Available from: 2022. 7 August 2024 https://pollen.aaaai.org/#/pages/about-the-nab.Accessed.

[17] Addison-Smith B., Wraith D.C., Davies J.M. (2020). Standardising pollen monitoring: quantifying confidence intervals for measurements of airborne pollen concentration. Aerobiologia.

[18] Milic A., Addison-Smith B., Van Haeften S., Davies J.M. (2021). Analysis of quality control outcomes of grass pollen identification and enumeration: experience matters. Aerobiologia.

[19] Sofiev M., Berger U., Prank M. (2019). MACC regional multi-model ensemble simulations of birch pollen dispersion in Europe. Atmos Chem Phys.

[20] Lo F., Bitz C.M., Hess J.J. (2021). Development of a Random Forest model for forecasting allergenic pollen in North America. Sci Total Environ.

[21] Bannister T., Ebert E., Silver J. (2021). A pilot forecasting system for epidemic thunderstorm asthma in south-eastern Australia. Bull Am Meteorol Soc.

[22] Emmerson K.M., Addison-Smith E., Ebert E. (2022). Evaluation of the performance of short-term curated daily airborne grass pollen forecasts in diverse biogeographical regions during the AusPollen Partnership project 2016–2020. Atmos Environ X.

[23] Anderegg W.R.L., Abatzoglou J.T., Anderegg L.D.L., Bielory L., Kinney P.L., Ziska L. (2021). Anthropogenic climate change is worsening North American pollen seasons. Proc Natl Acad Sci USA.

[24] Addison-Smith B., Milic A., Dwarakanath D. (2021). Medium-term increases in ambient grass pollen between 1994-1999 and 2016-2020 in a subtropical climate zone. Front Allergy.

[25] Ziska L.H., Makra L., Harry S.K. (2019). Temperature-related changes in airborne allergenic pollen abundance and seasonality across the northern hemisphere: a retrospective data analysis. Lancet Planet Health.

[26] Beggs P.J., Zhang Y., McGushin A. (2021). The 2021 report of the MJA-Lancet Countdown on health and climate change: Australia increasingly out on a limb. Med J Aust.

[27] Pawankar R., Wang J.Y., Wang I.J. (2020). Asia pacific association of allergy asthma and clinical immunology white paper 2020 on climate change, air pollution, and biodiversity in asia-pacific and impact on allergic diseases. Asia Pac Allergy.

[28] Buters J.T.M., Antunes C., Galveias A. (2018). Pollen and spore monitoring in the world. Clin Transl Allergy.

[29] Sauliene I., Sukiene L., Daunys G. (2019). Automatic pollen recognition with the Rapid-E particle counter: the first-level procedure, experience and next steps. Atmos Meas Tech.

[30] Miki K., Kawashima K. (2021). Estimation of pollen counts from light scattering intensity when sampling multiple pollen taxa – establishment of an automated multi-taxa pollen counting estimation system (AME system). Atmos Meas Tech.

[31] Tummon F., Adamov S., Clot B. (2021). A first evaluation of multiple automatic pollen monitors run in parallel. Aerobiologia.

[32] Sofiev M., Buters J., Tummon F. (2023). Designing an automatic pollen monitoring network for direct usage of observations to reconstruct the concentration fields. Sci Total Environ.

[33] Kawashima S., Thibaudon M., Matsuda S. (2017). Automated pollen monitoring system using laser optics for observing seasonal changes in the concentration of total airborne pollen. Aerobiologia.

[34] Clot B., Gilge S., Hajkova L. (2020). The EUMETNET AutoPollen Programme: establishing a prototype automatic pollen monitoring network in Europe. Aerobiologia.

[35] Morton S., Pencheon D., Bickler G. (2019). The sustainable development goals provide an important framework for addressing dangerous climate change and achieving wider public health benefits. Publ Health.

[36] Levetin E., Pityn P.J., Ramon G.D. (2023). Aeroallergen monitoring by the national allergy Bureau: a review of the past and a look into the future. J Allergy Clin Immunol Pract.

[37] Davies J.M., Berman D., Beggs P.J. (2021). Global climate change and pollen aeroallergens: a southern hemisphere perspective. Immunol Allergy Clin.

[38] Braun V., Clark V. (2006). Using thematic analysis in psychology. Qual Res Pychol.

[39] Abraham V., Hicks S., Svobodová-Svitavská H. (2021). Patterns in recent and Holocene pollen accumulation rates across Europe - the Pollen Monitoring Programme Database as a tool for vegetation reconstruction. Biogeosciences.

[40] National Health and Medical Research Council (2023). National Health and Medical Research Council.

[41] Esterhuizen N., Berman D.M., Neumann F.H. (2023). The South African pollen monitoring network: insights from 2 years of national aerospora sampling (2019-2021). Clin Transl Allergy.

[42] Ramon G.D., Vanegas E., Felix M. (2020). Year-long trends of airborne pollen in Argentina: more research is needed. World Allergy Organ J.

[43] Davies J.M., Smith B.A., Milic A. (2022). The AusPollen partnership project: allergenic airborne grass pollen seasonality and magnitude across temperate and subtropical eastern Australia, 2016-2020. Environ Res.

[44] Publications Office of the European Union (2017). Law E-LAtE.

[45] Hussinger K., Carvalho J.N. (2022). The long-term effect of research grants on the scientific output of university professors. Ind Innovat.

[46] Digital Science, Hahnel M., Smith G., Scaplehorn N., Schoenenberger H., Day L. (2023). The State of Open Data 2023: the longest-running longitudinal survey and analysis on open data.

[47] Chubb J., Watermeyer R. (2017). Artifice or integrity in the marketization of research impact? Investigating the moral economy of (pathways to) impact statements within research funding proposals in the UK and Australia. Studies Higher.

[48] Thelwall M., Simrick S., Viney I., van den Besselaar P. (2023). What is research funding, how does it influence research, and how is it recorded? Key dimensions of variation. Scientometrics.

[49] Hove K.H. (2020). Does the type of funding influence research results – and do researchers influence funders?. Prometheus.

[50] Nogrady B. (2022). Australian researchers push to end politicians' power to veto grants. Nature.

[51] Kmenta M., Zetter R., Berger U., Bastl K. (2016). Pollen information consumption as an indicator of pollen allergy burden. Wien Klin Wochenschr.

[52] Publishing O., Organisation for Economic Co-operation and Development (2023). Part-time Employment Rate.

[53] Schell S.F., Luke D.A., Schooley M.W. (2013). Public health program capacity for sustainability: a new framework. Implement Sci.

[54] Walker B., Crépin A.S., Nyström M. (2023). Response diversity as a sustainability strategy. Nat Sustain.

[55] Baldwin L., Flemming M.L., Baldwin L. (2020). Health Promotion in the 21st Century: New Approaches to Achieving Health for All.

[56] Cosma S., Rimo G., Cosma S. (2023). Conservation finance: what are we not doing? A review and research agenda. J Environ Manag.

[57] Kavanagh S., Shiell A., Hawe P., Garvey K. (2020). Resources, relationships, and systems thinking should inform the way community health promotion is funded. Crit Publ Health.

[58] Thien F., Beggs P.J., Csutoros D. (2018). The Melbourne epidemic thunderstorm asthma event 2016: an investigation of environmental triggers, effect on health services, and patient risk factors. Lancet Planet Health.

[59] Colas C., Brosa M., Anton E. (2017). Estimate of the total costs of allergic rhinitis in specialized care based on real-world data: the FERIN Study. Allergy.

[60] Walker S., Khan-Wasti S., Fletcher M., Cullinan P., Harris J., Sheikh A. (2007). Seasonal allergic rhinitis is associated with a detrimental effect on examination performance in United Kingdom teenagers: case-control study. J Allergy Clin Immunol.

[61] National Centers for Environmental Information (2021). https://www.ncdc.noaa.gov/sotc/global/202107.

[62] Estacio E.V., Oliver M., Downing B., Kurth J., Protheroe J. (2017). Effective partnership in community-based health promotion: lessons from the health literacy partnership. Int J Environ Res Publ Health.

[63] Binskin M., Bennett A., Macintosh A. (2020). https://naturaldisaster.royalcommission.gov.au/publications/html-report/introduction.

[64] Davies J.M., Nriagu J. (2019). Encyclopedia of Environmental Health.

