# Supplementary material for: A global survey addressing sustainability of pollen monitoring
Source: World Allergy Organ J. 2024 Nov 19;17(12):100997. doi: 10.1016/j.waojou.2024.100997 (PMC11612360; doi:10.1016/j.waojou.2024.100997)
Supplement: Multimedia component 1 [file mmc1.docx]

**A global survey addressing sustainability of pollen monitoring**

Supplemental Material

**Supplemental appendix - Questionnaire**

(Identifying information indicated by <brackets> has been removed)

**Investigation of the Sustainability of Pollen Monitoring networks (SPM) Questionnaire**

Participant Information

Please indicate your consent to help us investigate the sustainability of pollen monitoring networks globally by clicking **NEXT below.** This SPM study aims to find out how pollen monitoring networks are managed and sustained globally. The objective is to identify the characteristics of pollen monitoring networks globally, including information on the types of organisations that manage and fund pollen monitoring stations and the employment status of people who operate the pollen monitoring stations. The knowledge generated in this study will provide new understandings to inform strategies to sustain the pollen monitoring networks, such as the < Network>, into the future.

As an individual, you are free to choose whether to participate or not in this study. The study has been approved by the <University> Human Research Ethics Committee (<approval number>).

We will aggregate questionnaire response data from all the pollen monitoring stations globally and use it only for academic research. No identifiable information will be published. Outcomes of this research will provide insights to develop a sustainable approach to delivering continuous pollen monitoring information to the community into the future. It takes *5 to 10 minutes* approximately to complete this questionnaire. *Participation is optional.*

This research is funded by <University> via a < Scholarship> for <first author’s> PhD research project.

If you would like to know more about the study, please contact <PhD or lead researcher by email>

**NEXT**

|  | **About pollen monitoring in your country** |
| --- | --- |
| 1 | Which country is your pollen monitoring network/station located? (text) |
| 2 | How many pollen monitoring stations are in your country? (integer, number) |
| 3 | Is your pollen monitoring station part of a network?  (check box) Yes/No/I don’t know |
| 4 | If yes, then “how many pollen monitoring stations are in your network? (number, integer) |
|  | **About your pollen monitoring network** |
| 5 | How many pollen monitoring stations are in your network? (integer, number) |
| 6 | How long in years has the longest running station in your network been running? (integer, years) |
| 7 | How long has the newest station in your network been running? |
| 8 | When was the pollen monitoring network setup? (integer, year) |
| 9 | Is the network you coordinate part of a regional or national network? (Please tick one or mor that apply)  Regional  National  Other, please specify |

|  | **About your pollen monitoring station** |
| --- | --- |
| 10 | What organisation(s) undertakes the pollen monitoring (tick one or more that apply most appropriately to your network/station)   - 1. University   2. Research institute   3. Government agency      1. meteorology      2. air quality   4. hospital or health service      1. Private      2. Public   5. non-government organisation      1. Foundation supporting allergy patients      2. Foundation supporting asthma patients   6. Company or business   7. Other, please specify |
| 11 | What instrument do you use to monitor pollen? (please tick one or more that apply)   1. Hirst-type pollen trap (Burkard/Lanzoni) 2. Rotorod pollen trap 3. Automatic pollen monitor 4. Other, please specify |
| 12 | Are the station(s) you coordinate visited on a daily or weekly basis to collect the monitored sample?   1. Daily 2. Weekly 3. Both (Daily or weekly, depending on season) |
| 13 | What period of the year does your station(s) conduct aerobiological monitoring?  a. Annually (full duration of the year)  b. Seasonally |

|  | **About sharing pollen information** |
| --- | --- |
| 14 | During the pollen season, do the station(s) you coordinate share aerobiological information every day?  Yes/ No |
| 15 | If you communicate pollen information to people in your community, how is pollen information shared?   1. Directly to community via social media apps 2. Via counting organisation webpage 3. Via external organisation (e.g. Foundation, meteorology or air quality organisation) 4. Other, please specify |
|  | **About the people monitoring pollen** |
| 16 | How many pollen counters contribute to collecting samples and counting pollen at your network/station? (Specify number)   1. collecting the sample from the trap (integer, number) 2. counting the pollen (integer, number) |
| 17 | Who contributes to collecting and counting pollen at your station(s)? (please tick one or more that apply)   - 1. Students      1. Bachelor      2. Masters      3. PhD   2. Employees      1. Part time      2. Casual      3. Full time   3. Volunteers   4. Other, please specify |
| 18 | For the pollen counters at your site, is pollen monitoring _______ (please tick one or more that apply)   1. The primary task or duty of the role 2. Part of the core duties or usual role 3. Additional to core duties or usual role 4. Other, please specify |

|  | **About the funding sources for sustaining your pollen monitoring network** |
| --- | --- |
| 19 | How is pollen monitoring supported for the sites you coordinate? (Explain your chosen option in the open text box or elaborate you answer in the subsequent questions 20-23)   1. Unfunded- in kind contribution 2. Competitive government research project grant funding 3. Other short-term research grants 4. Direct university funding 5. Government agency funding    - 1. Health      2. Air quality      3. Meteorology      4. Environment    1. Other, please explain _______________ |
| 20 | If you receive grants to support pollen monitoring, then what type of grant scheme have you received funding from? (please choose all that apply)   1. Partnership or grants requiring co-contributions from other sources 2. Project 3. Program or multi-centre grant 4. Scholarship 5. Prefer not to answer 6. Other, please specify _______________ |
| 21 | \| If you receive grant funding to support pollen monitoring, what is the duration of current grant funding?  (Please indicate the first and the last year of competitive grant funding) \| \| --- \| |
| 22 | If your pollen monitoring station or network is supported by university funding, what is the duration of funding?  (Please indicate the first and last year of university funding) |
| 23 | If your pollen monitoring station/network is supported by government agencies, then please indicate the duration of funding  (Please indicate the first and last year of government funding) |

|  | About your pollen monitoring information |
| --- | --- |
| 24 | Why do you monitor pollen? (Open answer question) |
| 25 | Is pollen concentration data used for research purposes? (tick all that apply)   1. Research on pollen forecasting 2. Research on health impacts 3. Research on ecological impact 4. Research on climate change 5. Other research, please state ___________________ |
| 26 | How many pollen types do you count? (Specify number) |
| 27 | How many fungal spore types do you count? (Specify number) |
| 28 | What do you consider to be the pollen taxa that has the most impact on human health at your location? |
|  | Other comments or feedback about sustaining pollen monitoring |
| 29 | Do you have any other comments/feedback about the questionnaire or about sustaining pollen monitoring? |

*Please click SUBMIT to finish.*

**SUBMIT**

*Thank you for taking the time to contribute to this research.*

The summary of the findings of the SPM Questionnaire will be submitted for publication and can be made available upon request. If you require further information, please email


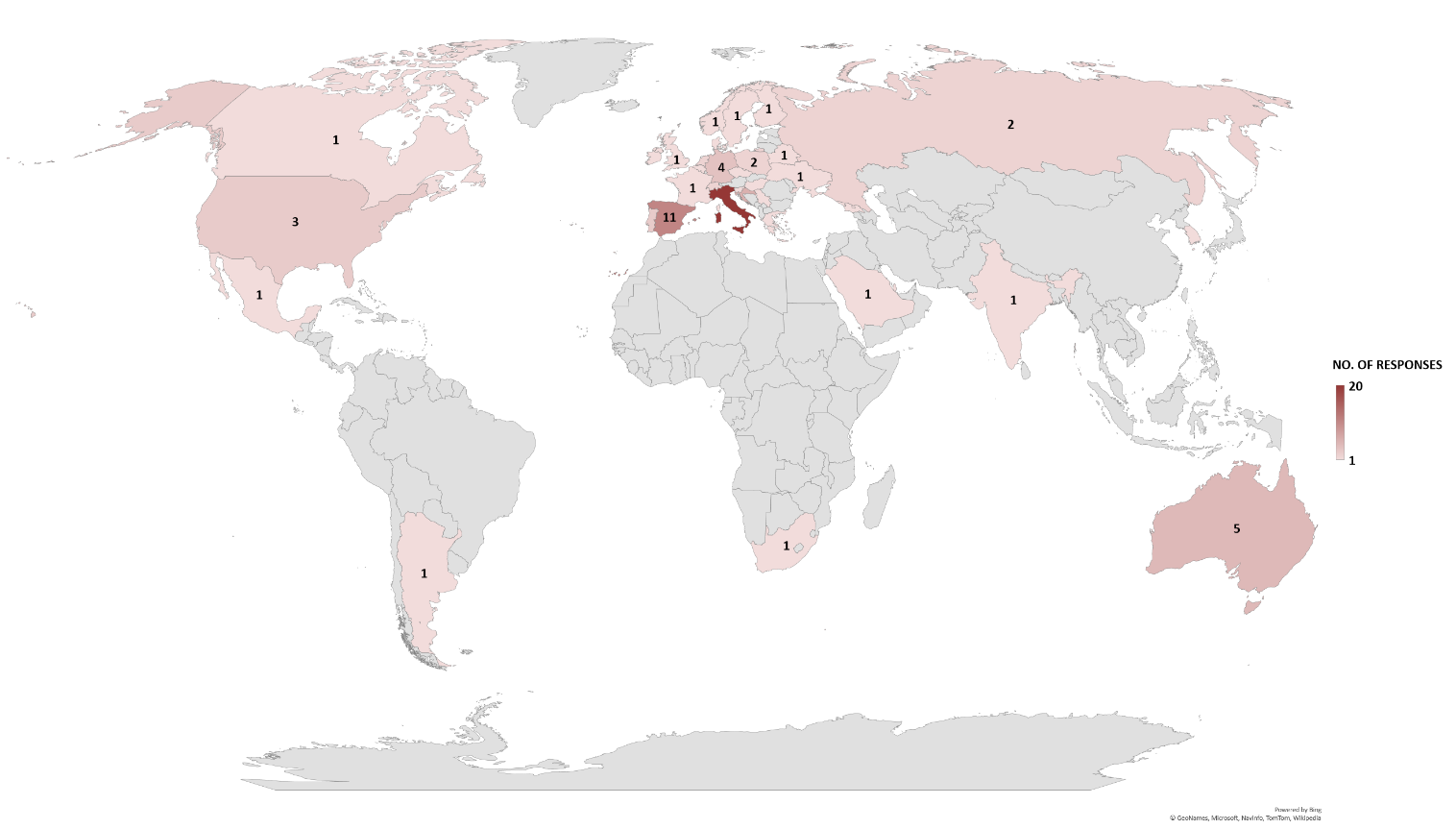


**Supplemental Figure 1. Number of responses to Sustaining Pollen Monitoring Questionnaire from each country.** All participating countries are in shades of red depending on the number of responses from participants in each country. Darker shades the of Red indicate a Higher the Participation. There were no responses from countries in Grey.

**Supplemental Table 1. Overarching and sub-themes and exemplary references in response to the open answer question “Why pollen is monitored?”**

| **OVERARCHING THEME** | **SUB-THEME (NUMBER OF REFERENCES)** | **REFERENCE EXAMPLE** |
| --- | --- | --- |
| CLIMATE CHANGE (EFFECT ON POLLEN) | Climate change (8) | “…to investigate climate changes…” |
|  | Climate change effects on pollen (6) | “…to study climatic changes related to pollen…”  “…evaluation of the effects of climate change on the beginning and end of flowering and the quantities of pollen…” |
| JOB AND DUTY | (9) | “Because it is my task at the university…”  “It is my job…”  “Initially it was a part of my research project, now it is a social duty.” |
| AEROBIOLOGY AND PREDICTION OF AIRBORNE POLLEN INFORMATION | Pollen abundance; levels; concentrations (8) | “… to make basic research on pollen abundance…”  “…to give rapid information about the levels of airborne allergenic pollen…” |
|  | Pollen seasonality (6) | "…to make basic research on pollen abundance and seasonality…"  "...evaluation of the effects of climate change on the beginning and end of flowering …" |
|  | Pollen calendars (2) | “Generate a pollen calendar…” |
|  | Pollen forecast; risk alerts; bulletin; app; (11) | “Monitor pollen levels to be able to produce pollen forecasts.”  “To provide real-time operational allergy risk alerts…”  "…pollen information is used as "goodie" in the App which is intended to collect symptoms over time from pollen allergic people. " |
|  | Pollen transport (2) | “…monitoring of transboundary pollen transport through air masses.” |
|  | Types of pollen (3) | “Our aims…the type of pollen present in the air.” |
| CLINICAL ALLERGY AND ENVIRONMENTAL PUBLIC HEALTH | Preventative measures; health protection (5) | “We monitor pollen as part of our Program of preventive action on health protection.”  “In order to protect human health by giving allergy sufferers the knowledge they need to minimise exposure.” |
|  | Support to medical and health services (16) | “We want to make pollen and fungal spore findings available to all Health Care Professionals…”  “…To inform allergy sufferers and medical/health services…” |
|  | Support to allergy patients (20) | “As a service to persons suffering from pollen allergies.”  “…Education to patients and families…”  “…to inform allergy sufferers…” |
|  | Allergy diagnosis; prognosis; medication use; treatment (5) | "…to optimize the providing of drugs during the pollen season."  "To improve the diagnosis and treatment of patients with pollinosis." |
|  | Association with symptoms (2) | "We work on solutions that help allergic persons to reduce their symptoms." |
| RESEARCH | (19) | “Research purposes (allergy, phenology, urban landscape).”  “We also collaborate with climate change researchers, environmental health specialists, public health surveys and projects, researchers in health impacts, statisticians who work with climate stats and plant invasion and agricultural researchers.” |
| OTHER | Biodiversity (2) | "…for studies on biodiversity..." |
|  | Allochthonous plants (7) | “…monitoring the spread of allochthonous plants…” |
|  | Fungal spores (7) | “…monitor other airborne particles such as fungal spores.” |
|  | Air pollution (2) | “…try to find a bound between air pollution, human health and biomaterial suspended in the air…” |

**Supplemental Table 2. Overarching and sub-themes and exemplary references in responses to the open answer question on “Other Feedback on the Questionnaire or Suggestions to Sustain Pollen Monitoring”.**

| OVERARCHING THEME | SUB-THEME (NUMBER OF REFERENCES) | REFERENCE EXAMPLE |
| --- | --- | --- |
| SUSTAINABILITY OF POLLEN MONITORING | Financial burden of pollen monitoring (5) | “Not being funded currently as there are no funds to support this. We are just doing this to maintain the stations on our own.”  "Without funding, it is difficult to maintain a pollen monitoring station…” |
|  | Impact of the pandemic (2) | "This year 2020, due to the pandemic and the economic crisis derived from it, it is likely that the entire network will stop working, and only a few stations will remain, but without a common project between them…” |
|  | Cessation of pollen monitoring (3) | "The monitoring was stopped in 2016 because of funding for a research assistant was not available…” |
|  | Costs in pollen monitoring/ maintenance (3) | “…with respect to automatic pollen monitoring...the costs, or cost savings. Thus, investments in pollen measurement can continue to be justified automatically or traditionally."  "...The type of climate and other environmental factors in the country also hastens the "wear and tear" of the pollen trap used…." |
|  | Role of government in funding, or (lack of) government responsibility (4) | "Sustainability is a major issue in <country> as governments typically do not see sustaining pollen monitoring as core business…”  “…it is insecure and a long-term sustainable model such as funding via the <meteorological agency> would be ideal." |
|  | Administrative load (2) | “…the main difficulty is dealing individually with each of the parties for many different aspects: the annual reporting, negotiating the amount and duration of grants, responding to specific requests, etc.” |
| TRAINING AND QUALITY OF POLLEN DATA | Training of pollen counters; comparability of data (3) | “More care should be given to data quality, especially to focal points such as microscope slide reading surface, reading approach and operator training, in order to obtain comparable data.” |
|  | Need for standardisation in pollen monitoring (2) | “Higher standardization of aerobiological monitoring activities.” |
|  | Quality of data (6) | "Give maximum attention to the quality of the data."  "More attention to data quality."  ‘…. improve the % <area> of reading” |
| RESEARCH AND COLLABORATION | National research projects (1) | “We are part of a project funding a national project of four stations in four states but contribute to coordination of other sites…Our University directly manages four sites run by our research group for three projects and contributes to a broader national network of 25 sites and multiple projects.” |
|  | Relevance/ representativeness (1) | "My devices are not representative for <country>, the real network is manual and is governed by University of <city>, who are also responsible for the allergy risk forecasts." |
|  | Collaboration and sharing (3) | "We collaborate with the <name>-network, the network of the <country> Environment Protection Agencies, furthermore our data are sent to the <continent>-database." |
|  | Centralisation (1) | "The main question is to centralize pollen counting or not? In the case of centralization, shipping costs are high, but we need fewer workers…” |
| AUTOMATIC REAL-TIME POLLEN MONITORING | (3) | “I am convinced that automatic real-time pollen identification is the way forward…” |
| VALUE OF POLLEN MONITORING | (4) | “Pollen monitoring is necessary for allergy diagnosis and for population level allergic sensitivities."  “We believe that every effort to continue with the monitoring network is excellent and worthwhile…” |
| FEEDBACK ON STUDY | (4) | “Well done questionnaire!”  “…the topic of research is very interesting. I look forward to the results.” |

Worldwide Map of Pollen Monitoring Stations

**This interactive map is a result of EAACI Task Force TF-40108**
**‘Inventory of pollen monitoring sites in the world’**

Contributors:
Project managers: Celia Antunes, Jeroen Buters
Database building: Ana Galveias, Celia Antunes, Jose Oteros, Jeroen Buters
Interactive map development and administrator: Jose Oteros
Members of the Task Force 40108 (Aerobiology and Air Pollution): C. Antunes, M. Thibaudon, A. Galveias, J. Oteros, C. Galan, M. Werchan, and J. Buters

Website options

https://patients.eaaci.org/worldwide-map-of-pollen-monitoring-stations/

[https://www.zaum-online.de/pollen/pollen-monitoring-map-of-the-world/index.html](https://urldefense.com/v3/__https:/www.zaum-online.de/pollen/pollen-monitoring-map-of-the-world/index.html__;!!NVzLfOphnbDXSw!G7C1_sJVy-IOxxc1wJGeYFVvaX058jvUXdsbY1oObMGICEyUuw3xjJKqmjlCQ8hLHlI2c5bhydzIKzStlRMK$)

Option to contribute a new pollen monitoring station

Individuals who coordinate a pollen monitoring station that is new, or not included on this map, can now add a new station themselves.

See About Map and “Contact Us” information

https://www.zaum-online.de/pollen/pollen-monitoring-map-of-the-world/index.html

A

B

**Supplementary Figure 2** A. Reported number of pollen and fungal spore taxa counted by sites (n= 84 responses), and B. pollen taxa considered to have the most impact on human health at the stie location (open text answered by all 84 respondents).
